# An LC–MS-based designated comparison method with similar performance to the Lp(a) reference measurement procedure to guide molar Lp(a) standardization

**DOI:** 10.1186/s12014-023-09446-5

**Published:** 2024-01-24

**Authors:** Nina M. Diederiks, L. Renee Ruhaak, Fred P. H. T. M. Romijn, Mervin M. Pieterse, Nico P. M. Smit, Christa M. Cobbaert

**Affiliations:** Department of Clinical Chemistry and Laboratory Medicine, Leiden University Medical Center, Postzone E2-P, Albinusdreef 2, 2333 ZA Leiden, The Netherlands

## Abstract

**Background:**

The 2022 consensus statement of the European Atherosclerosis Society (EAS) on lipoprotein(a) (Lp(a)) recognizes the role of Lp(a) as a relevant genetically determined risk factor and recommends its measurement at least once in an individual’s lifetime. It also strongly urges that Lp(a) test results are expressed as apolipoprotein (a) (apo(a)) amount of substance in molar units and no longer in confounded Lp(a) mass units (mg/dL or mg/L). Therefore, IVD manufacturers should transition to molar units. A prerequisite for this transition is the availability of an Lp(a) Reference Measurement Procedure (RMP) that allows unequivocal molecular detection and quantification of apo(a) in Lp(a). To that end an ISO 17511:2020 compliant LC–MS based and IFCC-endorsed RMP has been established that targets proteotypic peptides of apolipoprotein(a) (apo(a)) in Lp(a). The RMP is laborious and requires highly skilled operators. To guide IVD-manufacturers of immunoassay-based Lp(a) test kits in the transition from mass to molar units, a Designated Comparison Method (DCM) has been developed and evaluated.

**Methods:**

To assess whether the DCM provides equivalent results compared to the RMP, the procedural designs were compared and the analytical performance of DCM and RMP were first evaluated in a head-to-head comparison. Subsequently, apo(a) was quantified in 153 human clinical serum samples. Both DCM and RMP were calibrated using external native calibrators that produce results traceable to SRM2B. Measurement uncertainty (MU) was checked against predefined allowable MU.

**Results:**

The major difference in the design of the DCM for apo(a) is the use of only one enzymatic digestion step. The analytical performance of the DCM and RMP for apo(a) is highly similar. In a direct method comparison, equivalent results were obtained with a median regression slope 0.997 of and a median bias of − 0.2 nmol/L (− 0.2%); the intermediate imprecision of the test results was within total allowable error (TEa) (CVa of 10.2% at 90 nmol/L).

**Conclusions:**

The semi-automated, higher throughput, LC–MS-based method for Lp(a) meets the predefined analytical performance specifications and allowable MU and is hence applicable as a higher order Designated Comparison Method, which is ideally suited to guide IVD manufacturers in the transition from Lp(a) mass to molar units.

## Introduction

The 2022 expert consensus by the European Atherosclerosis Society (EAS) recommends that in all individuals an assessment of lipoprotein (a) (Lp(a)) should be conducted alongside the standard serum lipid profile at least once in a lifetime [1, 2]. While Lp(a) as a risk factor for cardiovascular disease has long been controversial, these recommendations are now based on updated evidence for a causal continuous association in different ethnic groups between Lp(a) concentrations and adverse cardiovascular outcomes [1]. The EAS expert consensus moreover advises that Lp(a) test results are expressed as apo(a) amount of substance in molar units -due to extreme apo(a) heterogeneity- and no longer in confounded Lp(a) mass units (mg/dL or mg/L). With these recommendations, as well as the promise of Lp(a) lowering drugs [1, 3–6], the need for accurate quantification of Lp(a) in molar units is now urgent.

Currently, many IVD manufacturers market kits that provide results in mass units even though standardization and expression of Lp(a) measurements in molar units have been recommended for > 2 decades [1, 7–9]. Flawed Lp(a) results should be overcome and therefore IVD manufacturers must transition from mass units to molar units. A prerequisite for this transition is the availability of an Lp(a) Reference Measurement Procedure (RMP) that allows unequivocal molecular detection and quantification of apolipoprotein(a) (apo(a)) in Lp(a). The International Federation for Clinical Chemistry and Laboratory Medicine (IFCC) working group for standardization of apolipoproteins by mass spectrometry (IFCC WG APO-MS) set out a conceptual approach for such an RMP [8], and recently reported on an ISO 17511:2020 compliant liquid chromatography – mass spectrometry (LC–MS) based and IFCC-endorsed RMP [10]. The RMP quantifies proteotypic peptides of apo (a) which is the specific apolipoprotein and a major structural component of lipoprotein(a) [11]. Importantly, apo(a) is a highly heterogenic protein that manifests in various proteoforms, due to the KIV_2_ polymorphisms and post translational modifications [10, 12]. Moreover, it is present in serum in a wide concentration range. As MS allows for quantification of molecularly defined peptides, the apo(a) peptides were selected to accurately reflect the intended measurand (apo(a) amount of substance in molar units).

A RMP is a higher order measurement procedure in the metrological traceability chain, as outlined in ISO 17511:2020 [13]. Therefore, the method has to be of high quality and execution of the procedure should take place according to stringent quality measures as outlined in ISO 15195:2018 [14]. Such high quality and confidence in measurement accuracy, however, comes at the cost of ease of use of the procedure. To facilitate the transition from mass to molar units, a Designated Comparison Method (DCM) would be beneficial. To that end, a semi-automated LC–MS based method for quantitation of apo(a) in molar units was developed; the method is easier to operate and comprises only a single proteolytic digestion step.

To ensure the method is suited for its intended purpose, the DCM should produce equivalent results and have a comparable measurement uncertainty as the RMP. Its total error budget should only be a fraction of the allowable error budget (TEa), as deduced from biological variation. This allowable error is set at the level of the end user, but is divided over the various stakeholders/methods in the traceability chain. We therefore propose that the DCM may not consume more than 1/2 of the total allowable error (TEa) budget. For trueness, the correlation coefficient of a comparison between the two methods should be > 0.975, and the slope of the comparison may not significantly deviate from 1.000 under the used similar external calibration procedure. In this study, we performed a head-to-head comparison between the RMP and the DCM, both at the levels of their design and analytical performance, to ensure results generated with the DCM can be used to guide IVD manufacturers in their transition towards nmol/L units.

## Materials and methods

This section briefly describes the semi-automated DCM compared to the previously published RMP [15]. Subsequently, the method comparison is described. Common solvents and consumables are described at the start of this section.

### Materials

Sequencing grade modified trypsin (V5111) and mass spectrometry grade Lys-C (VA1170) were acquired from Promega (Leiden, The Netherlands). LC–MS grade methanol (MeOH) was purchased from Biosolve (Valkenswaard, The Netherlands) and formic acid (FA, ≥ 99% purity) was purchased from Avantor/VWR (Radnor, PA, USA). Ammonium bicarbonate (ABC) was purchased from Fluka (Landsmeer, the Netherlands) while Iodoacetamide (IAM), Sodium Deoxycholate (DOC) and tris(hydroxymethyl)aminomethane (TRIS), pH 8.1, were purchased from Sigma-Aldrich (Zwijndrecht, The Netherlands). Tris(2-carboxyethyl)phosphine (TCEP) was purchased from Thermofisher Scientific (Waltham, MA). Synthetic and stable isotope labelled peptides were produced by our in-house facility. The assessment of their purity was conducted using LC with UV detection and matrix assisted laser desorption/ionization (MALDI)-MS. Deidentified serum samples for calibration and quality control were procured from MCA Laboratory (Winterswijk, The Netherlands).

VACUETTE^®^ Secondary 13 × 75 mm MULTIPLEX PET tubes (459011), U-bottom (650201), V-bottom 96 well microplates (651201), plate lids (656102), 15 mL Cellstar tubes (188271) and 50 mL Cellstar tubes (227261) were from Greiner bio-one (Alphen aan den Rijn, the Netherlands). Skirted (0030.128.680) and semi-skirted (0030.128.613) 96-well PCR plates were from Eppendorf (Nijmegen, the Netherlands). Bioke (Leiden, the Netherlands) supplied gas permeable moisture barrier seals (4ti-0516/96) and pierceable seals (4ti-0566). 250 µL Tips for Bravo (19477–002) were from Agilent Technologies (Santa Clara, CA, USA). and Oasis Prime HLB µElution Plates (186008052) were from Waters Corporation (Milford, PA, USA).

### Semi-automated LC–MS method for the quantitation of apo(a)

Apo(a) quantification was implemented in our already existing test for multiplexed apolipoprotein quantification and was conducted according to the standard operating procedure for sample preparation and tryptic digestion, as outlined previously [15, 16]. In short, Samples were 20 × diluted in 100 mmol/L TRIS, followed by protein solubilization and cysteine reduction using DOC and TCEP at 56 °C. Cysteine thiols were methylated by IAM at room temperature in the dark, immediately followed by trypsin digestion at a protein:enzyme ratio of 35:1 (w/w) at 37 °C. After 3 h, the reaction is quenched and peptides formed are enriched using Oasis HLB SPE, eluting the peptides with 55% MeOH. The workflow is semi-automated on a 96-channel BRAVO automated liquid handling platform (Agilent Technologies). The LC–MS system consisted of a 1290 multisampler (G7167B), and 1290 high speed analytical pump (G7120A) and 1290 multicolumn thermostat (G7116B), coupled to either a 6495A or 6495C triple quadrupole mass spectrometer. The instrument was operated in positive mode, in dynamic MRM mode with a cycle time of 500 ms. For apo(a), two peptides were monitored, each with three transitions. A method-specific system suitability test, comprising measurement of a mixture of synthetic and SIL peptides was performed along with each analytical run. An overview of the DCM and RMP method similarities and disparities can be found in Table 1.

**Table 1:** Head-to-Head comparison of the fundamental characteristics defining of the IFCC-endorsed Reference Measurement Procedure (RMP) and the Designated Comparison Method (DCM) for apo(a)

Method Characteristic	Reference Measurement Procedure	Designated Comparison Method
*General & Pre-analytical*		
Intended use	Higher order RMP as an essential part of the apo(a) traceability chain and future RMS	Designated Comparison Method for the future apolipoprotein RMS
Measurands addressed and units of reporting	Serum apo(a) (nmol/L)	Serum apo(a) (nmol/L)
Sample matrix	Serum	Serum
Automation	**No, Manual**	**Yes, Semi-automated**
Transition selection	Three transitions per peptide	Three transitions per peptide
Liquids applied in sample preparation	Buffer: **100 mM Ammonium Bicarbonate, pH 8.1** **Reduction mix: 1.15 mmol/L TCEP, 0.40% (v/v) DOC**	Buffer: **100 mM tris(hydroxymethyl)aminomethane, pH op 8.1** **Reduction mix: 0.5 mM TCEP, 0.523% (v/v) DOC**
Pre-digestion	**LysC, 1:700 w/w LysC-to-protein ratio**	**None**
Pre-digestion time	**1 h, 37 °C**	**N.A**
Digestion	Trypsin, **1:47** w/w Trypsin-to-protein ratio	Trypsin, **1:35** w/w trypsin-to-protein ratio
Digestion time	3 h, 37 °C	3 h, 37 °C
Main technology of sample purification	Solid phase extraction (off-line) with Oasis HLB 3 mg/well, eluted using 0.2 mL 80% MeOH	Solid phase extraction (off-line) with Oasis HLB 3 mg/well, eluted using 0.1 mL 55% MeOH
*Proteotypic peptides of apo(a)*		
Apo(a)	LFLEPTQADIALLK GISSTTVTGR **TPENYPNAGLTR**	LFLEPTQADIALLK GISSTTVTGR
*LC–MS acquisition conditions*		
General LC setup	Agilent 1290 infinity II ultra-high performance LC system	Agilent 1290 infinity II ultra-high performance LC system
Guard column	Zorbax SB-C18	Zorbax SB-C18
Guard column geometry	2.1 × 5 mm, 1,8 µm	2.1 × 5 mm, 1,8 µm
Analytical column	Zorbax SB-C18	Zorbax SB-C18
Main column geometry	2.1 × 50 mm, 1,8 µm	2.1 × 50 mm, 1,8 µm
Mobile phase constituents	MeOH (HPLC grade), FA, Ultrapure water	MeOH (HPLC grade), FA, Ultrapure water
Flow Rate	0.2 mL/min	0.2 mL/min
Sample injection Volume	10 μL	10 μL
Ionization Mode	Positive	Positive
total running window	20 min	19 min
Mass spectrometer	Agilent 6495A triple quadrupole mass spectrometer	Agilent 6495A & 6495C triple quadrupole mass spectrometer
Gradient, generic description	A: 5% MeOH and 0.05% FA in water; B: 95% MeOH and 0.05% FA in water. Starting condition: A 92% - Linear decrease to 82% A over 7 min—Linear decrease to 40% A at 15 min—Washing step: Steep decrease to 5% A at 15.1 min—Isocratic hold at 5% A until 17 min—Reequilibration: 3 min using starting conditions	A: 5% MeOH and 0.05% FA in water; B: 95% MeOH and 0.05% FA in water. Starting condition: A 95% - Linear decrease to 67% A over 8 min—Linear decrease to 43% A at 12 min—Washing step: Steep decrease to 5% A at 12.1 min—Isocratic hold at 5% A until 16 min—Reequilibration: 3 min using starting conditions
Main MS ionization mode	Electrospray, positive polarity	Electrospray, positive polarity
Fragmentation	Collision induced dissociation	Collision induced dissociation
*Run acceptance & quantitation*		
Internal Standard	In house (^13^C, ^15^N)R or (^13^C,^15^N)K SIL peptides	In house (^13^C, ^15^N)R or (^13^C,^15^N)K SIL peptides
Calibration concentrations	***MCA* 2013.2062** **apo(a): 93.4 nmol/L;**	***MCA* 2019.1564/2019.1565/2019.1566/2019.1568/2019.15611** **apo(a): 17.1 – 41.2 – 93.4 – 270.7 – 9.9 nmol/L;**
Type of calibration and calibration samples matrix	External protein calibration based on a native human serum sample with internal standard	External protein calibration based on native human serum samples with internal standard
Traceability	Indirect traceability to secondary reference material for apo(a): SRM-2B	Indirect traceability to secondary reference material for apo(a): SRM-2B
Data Analysis Software	Mass Hunter Workstation Quantitative/Qualitative Analysis software/Skyline	Mass Hunter Workstation Quantitative/Qualitative Analysis software
Interpretation of data	All transitions (both quantifying and qualifying) were evaluated individually	All transitions (both quantifying and qualifying) were evaluated individually

Major differences between the procedures are highlighted in bold

*Apo* apolipoprotein, *DCM* Designated Comparison Method, *RMP* Reference Measurement Procedure, *RMS* Reference Measurement System, *LC* Liquid Chromatography, *MS* Mass Spectrometry, *HPLC* High-Performance Liquid Chromatography, *TEa* Total Allowable Error, *WHO* World Health Organization, *IFCC* International Federation of Clinical Chemistry

### Reference Measurement Procedure for the quantitation of apo(a)

Apolipoprotein quantification was conducted according to the standard operating procedure, as outlined previously [10]. In short, samples were 20 × diluted in 100 mmol/L ABC. Proteins were solubilized by DOC and cysteines were reduced using TCEP at 56 °C. Cysteine thiols were methylated by IAM at room temperature in the dark, immediately followed by pre-digestion using Lys-C for 1 h at 37 °C at a protein:enzyme ratio of 700:1 (w/w). Then trypsin was added at a protein:enzyme ratio of 47:1 (w/w) and samples were further digested at 37 °C for 3 h. The reaction was quenched, and Oasis HLB SPE was employed for peptide enrichment, eluting the peptides with 80% MeOH. The workflow was performed manually. The LC–MS system consisted of a 1290 multisampler (G7167B), and 1290 high speed analytical pump (G7120A), and 1290 multicolumn thermostat (G7116B), coupled to a 6495A triple quadrupole mass spectrometer. The instrument was operated in positive mode, in dynamic MRM mode with a cycle time of 500 ms. For apo(a), three peptides were monitored, each with three transitions. A method-specific system suitability test, comprising measurement of a mixture of synthetic and SIL peptides was performed along with each analytical run. An overview of the method can be found in Table 1.

### Comparison between RMP and semi-automated Designated Comparison Method for apo(a)

A head-to-head comparison between the RMP and the DCM was performed based on their standard operating procedures and equivalence and analytical performance were evaluated according to CLSI EP-15. Moreover, analytical imprecision, as assessed through both a CLSI EP-15 protocol (measurement of five human serum samples in quintuplicate on five different days (total n = 25 per sample) and long-term IQC monitoring, were compared.

Deidentified Serum samples that were left over from general clinical chemistry analysis were collected from 153 human donors. In short, phlebotomy was conducted in Becton Dickinson (BD) serum gel tubes (367957) and blood was allowed to clot at room temperature for 30 min, followed by centrifugation at 3000 g for 8 min. The donors provided broad consent for the use of their deidentified biomaterial. An aliquot containing 250 μl was prepared immediately after centrifugation and stored at – 20 ℃ for up to 3 weeks. First, the DCM was performed in eight batches. After refreezing at − 20 °C for 3 to 7 months, the samples were analyzed with the RMP in two batches.

For data analysis, apo(a) concentrations below the limit of quantitation of < 3.8 nmol/L were excluded from method comparison analysis. Concentrations obtained by the DCM were plotted against the RMP results and linear regression was performed.

## Results

### Head-to-head comparison of the methods

Results of the head-to-head comparison of the RMP and the DCM are summarized in Table 1. While the procedures are highly similar and both based on the principle of bottom-up proteomics, differences between the procedure the facilitate ease of use of the DCM have been made. Specific differences between the methods are alternative peptide and transition selection, the use of a single calibrator (RMP) vs the use of five calibrators (DCM), the pre-digestion (RMP) and semi-automation (DCM). Each of these aspects are further elaborated below.

For the RMP, three proteotypic peptides are selected for quantitation: GISSTVTGR (kringle 5), LFLEPTQADIALLK (protease domain) and TPENYPNAGLTR (kringle 9). For the DCM, only two peptides are monitored: GISSTVTGR and LFLEPTQADIALLK. In both procedures three transitions are monitored per peptide; for peptide GISSTVTGR, the three transitions overlap, while for peptide LFLEPTQADIALLK, the b2 fragment is monitored in the RMP and the b3 fragment is monitored in the DCM (Table 2). The calibration of both the RMP and the DCM is in nmol/L and is currently based on native human serum calibrators that are indirectly traceable to WHO-IFCC reference material SRM2B. For the RMP a single point calibration is used (93.4 nmol/L), while 5 calibrators (17.1–270.7 nmol/L) are used in the DCM.

**Table 2:** Transitions monitored for the quantitation of apo(a) in both the RMP and the DCM

Peptide	SIL (y/n)	Precursor (m/z)	Quantifying fragment (m/z)	Qualifying fragment 1 (m/z)	Qualifying fragment 2 (m/z)
IFCC-endorsed Reference Measurement Procedure*					
GISSTTVTGR		489.8	808.4 (y8)	533.3 (y5)	634.4 (y6)
GISSTTVTG[R-U10]	Y	494.8	818.4	543.3	644.4
LFLEPTQADIALLK		786.5	1069.6 (y10)	261.2 (b2)	1198.6 (y11)
LFLEPTQADIALL[K + U8]	Y	790.5	1077.6	261.2	1206.6
TPENYPNAGLTR		666.8	728.4 (y7)	199.1 (b2)	891.5 (y8)
TPENYPNAGLT[R + U10]	Y	671.8	738.4	199.1	901.5
Designated Comparison Method					
GISSTTVTGR		489.8	808.4 (y8)	634.4 (y6)	533.3 (y5)
GISSTTVTG[R-U10]	Y	494.8	818.4	644.4	543.3
LFLEPTQADIALLK		786.5	1069.6 (y10)	1198.6 (y11)	374.2 (b2)
LFLEPTQADIALL[K + U8]	Y	790.5	1077.6	1206.6	374.2

^*^Data from Ruhaak et al. Clin Chem 2023[10]

Minor differences are present in the reagents used in the sample preparation: A 100 mM ABC buffer (pH 8.1) is used for the RMP, while a 100 mM TRIS buffer (pH 8.1) is used in the DCM. 1.15 mmol/L TCEP was used in the RMP as well as 0.40% (v/v) DOC and 4.6 mmol/L IAM. For the DCM 0.5 mM TCEP, 0.523% (v/v) DOC and 4.6 mmol/L IAM was used. A pre-digestion step using LysC is performed prior to trypsin digestion (1:47, w/w) in the RMP, while in the DCM trypsin digestion (1:35, w/w) is started immediately. SPE of the two procedures is performed on the HLB stationary phase, with elution using 80% MeOH in the RMP compared to 55% MeOH in the DCM. A major difference in the execution of the sample preparation is the semi-automation of the DCM, which is not yet implemented for the RMP.

### Analytical performance: intermediate imprecision

**Fig. 1:**
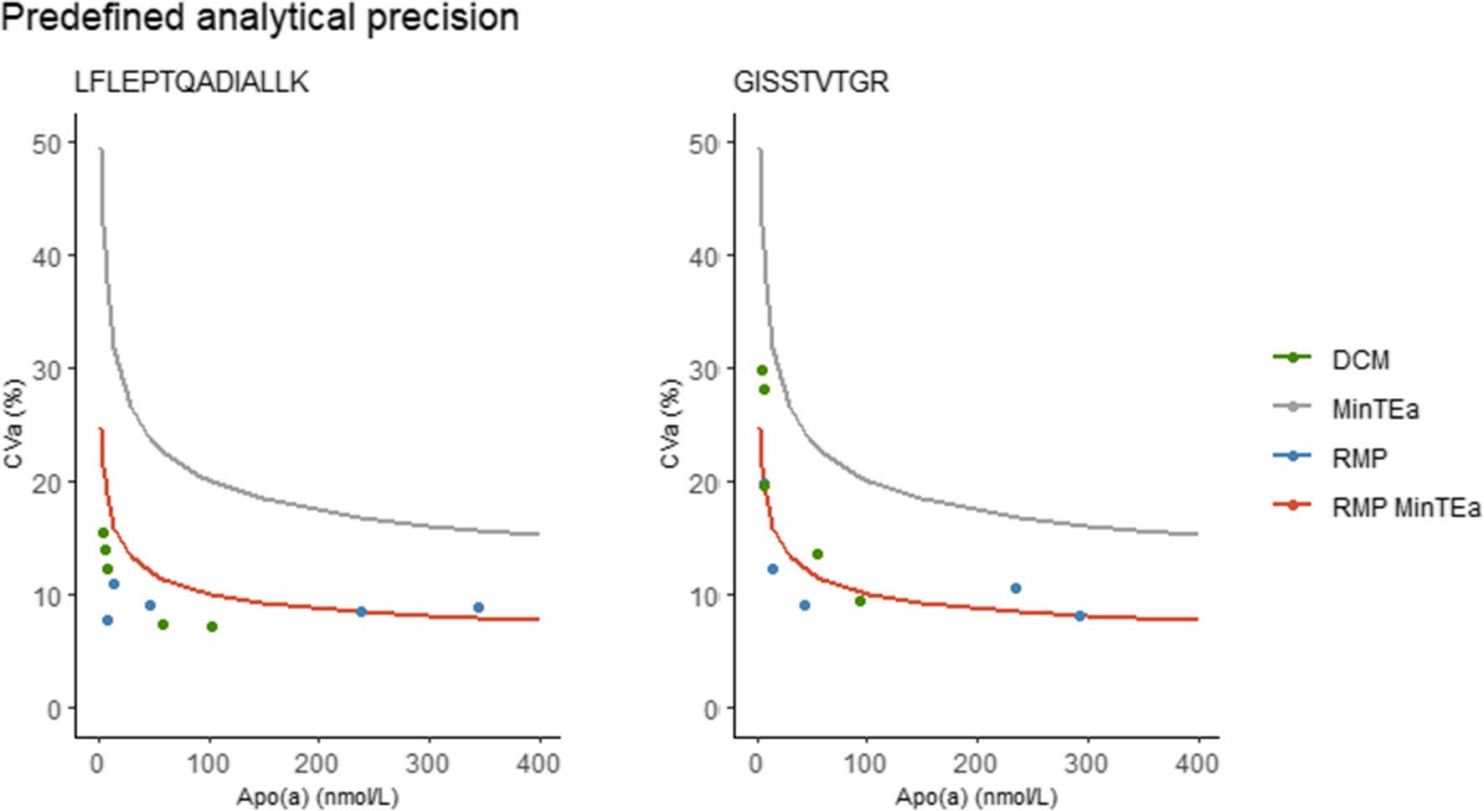
Comparison of imprecision of RMP and DCM based on CLSI EP-15 protocol results. Tests should fulfill predefined analytical performance specifications. For apo(a), the minimum total allowable error (TEa) is concentration dependent (grey line). To divide the error budget over the stakeholders of the metrological traceability chain, ½ of the TEa is allocated for the RMP, and thus ideally also the DCM (red line). EP-15 results are plotted as blue dots (RMP) or green dots (DCM)

While it is anticipated that the procedural differences between the RMS and the DCM will not cause deviating results, the analytical performances of both tests are compared. Medical tests should be fit-for-purpose. Therefore, their analytical performance specifications should be predefined. The allowable error budgets are set according to the Milan hierarchy [18], based ideally on clinical outcome, alternatively on biological variation data, and in other cases based on state-of-the-art. To the best of our knowledge, no data is available to defer error budgets for apo(a) based on clinical outcome studies; therefore, analytical performance specifications and total allowable error (TEa) based on biological variation are used. However, calculations typically rely on constant biological variation, independent of the analytes concentration. Yet, Lp(a) is a heterogenous particle containing the highly variable protein apo(a). This variability extends to both the concentration and structure of apo(a), including kringle IV-2 size polymorphism, as well as N- and O- glycosylation, leading to 1000-fold differences between individuals [12, 19–21]. The Lp(a) distribution is also racially dependent and does not follow a Gaussian curve for most of the races studied [21, 22]. Consequently, the intra- and inter-individual variation vary over the range of the Lp(a) concentrations. Indeed, a logarithmic intra-individual biological variation (CVi) was observed [23]. In error budget calculations, the logarithmic CVi can be considered. Measurements in Cobbaert et al. [23] were performed using a former state-of-the-art Lp(a) test, not yet standardized to the former Reference Measurement System (RMS) [23, 24]. However, assuming a (linear) conversion factor of 300 mg/L = 90 nmol/L, and a constant Inter-individual Coefficient of Variation (CVg) of 18.1%, a concentration dependent minimum TEa can be deduced (Fig. 1, grey line). The total error budget must be divided over the different stakeholders of the traceability chain and as such, the proportional error budget for the RMP was set at 50% (Fig. 1, red line).

To compare the analytical imprecision of the DCM with the RMP, the results of CLSI EP-15 protocols for evaluation of analytical imprecision (Table 3) are plotted in Fig. 1 (dots). For quantifying peptide LFLEPTQADIALLK, the analytical imprecision of the DCM fulfills the analytical performance specifications, while for qualifying peptide GISSTVTGR the imprecision is close to fulfillment of the analytical performance specifications. However, as the peptide is included for confirmation purposes, the criteria can be loosened.

**Table 3:** Results of an EP-15 analytical imprecision verification of both the RMP and the DCM

Sample	Peptide	Average (nmol/L)	CV wr (%)	CV br (%)	CV wl (%)	TEa (%)
Reference Measurement Procedure*						
1	GISSTVTGR	42.9	7.7	4.6	9.0	11.9
2	GISSTVTGR	7.4	14.4	13.6	19.8	18.7
3	GISSTVTGR	13.6	10.3	6.3	12.1	15.8
4	GISSTVTGR	234	8.1	6.7	10.5	8.4
5	GISSTVTGR	292	7	4.0	8.1	7.8
1	LFLEPTQADIALLK	46.7	7.3	5.0	8.9	11.9
2	LFLEPTQADIALLK	8	5.8	5.0	7.7	18.7
3	LFLEPTQADIALLK	14.9	8.8	6.5	10.9	15.8
4	LFLEPTQADIALLK	238	7.7	3.5	8.4	8.4
5	LFLEPTQADIALLK	344	6.6	5.7	8.7	7.8
Designated Comparison Method						
1	GISSTVTGR	4	29.7	0.0	29.7	21.3
2	GISSTVTGR	6	25.6	9.9	28.0	20.3
3	GISSTVTGR	7	19.6	0.0	19.6	18.7
4	GISSTVTGR	94	9.3	0.0	9.3	10
5	GISSTVTGR	55	13.5	0.0	13.5	11.3
1	LFLEPTQADIALLK	5	14.8	4.3	15.4	21.3
2	LFLEPTQADIALLK	6	13.8	0.0	13.8	20.3
3	LFLEPTQADIALLK	8	12.1	1.3	12.2	18.7
4	LFLEPTQADIALLK	102	5.9	3.7	7.0	10
5	LFLEPTQADIALLK	59	5.6	4.6	7.3	11.3

^*^Data from Ruhaak et al. Clin Chem 2023[10]

The long-term imprecision has also previously been monitored for both the RMP and the DCM. For the RMP, a native human serum IQC sample containing 44 nmol/L apo(a) yielded CVs of 9.0% and 10.0% for peptides GISSTVTGR and LFLEPTQADIALLK, respectively over a 2-year period, 84 measurements and 34 96-well batches [10]. Similar results have been obtained for the DCM, where a native human serum sample containing 102 nmol/L apo(a) yielded a CV of 7.3% for peptide LFLEPTQADIALLK over a 2-year period [16, 17]. Based on the comparison of both the CLSI EP-15 results as well as the long-term robustness, it can be concluded that the analytical performance of the semi-automated DCM is comparable to that of the RMP (Table 4)

**Table 4:** Summary statistics of IQC performance during method comparison

Peptide	QC1					QC2				
	Average concentration (nmol/L)	CV(%)	n	Target concentration (nmol/L)	Target CV(%)	Average concentration (nmol/L)	CV(%)	n	Target concentration (nmol/L)	Target CV(%)
Reference Measurement Procedure										
LFLEP	41	5.2	6	44	8.9	7	6.9	5	8	7.7
GISST	43	7.5	6	43	9.0	8	17.7	5	8	19.8
TPENY	38	8.2	6	41	13.4	8	5.5	5	8	23.3
Designated Comparison Method										
LFLEP	40	5.2	21	41	11.4	8	3.3	21	7	18.4
GISST	40	7.5	21	40	5.9	7	3.5	21	7	10.9

^*^Target concentrations and CVs were calculated based on longer-term evaluation of IQC performance

### Data validity for the method comparison

To define whether results obtained with the DCM are exchangeable with results obtained with the RMP, a method comparison was performed on 153 native human serum samples, each measured individually. First, measures of data validity (IQC, and assessment of intrinsic metadata) are presented, followed by the method comparison. The RMP results were generated in two batches, containing bilevel IQC (total n = 5). Average concentrations were 41 nmol/L and 7 nmol/L for QC1 and QC2 for peptide LFLEPTQADIALLK, with CVs of 5.2% and 6.9%. For the DCM, apo(a) was quantified in seven batches, each containing bilevel IQC (total n = 21). Average concentrations were 40 nmol/L and 8 nmol/L for QC1 and QC2 for peptide LFLEPTQADIALLK, with CVs of 5.2% and 3.3%.

**Fig. 2:**
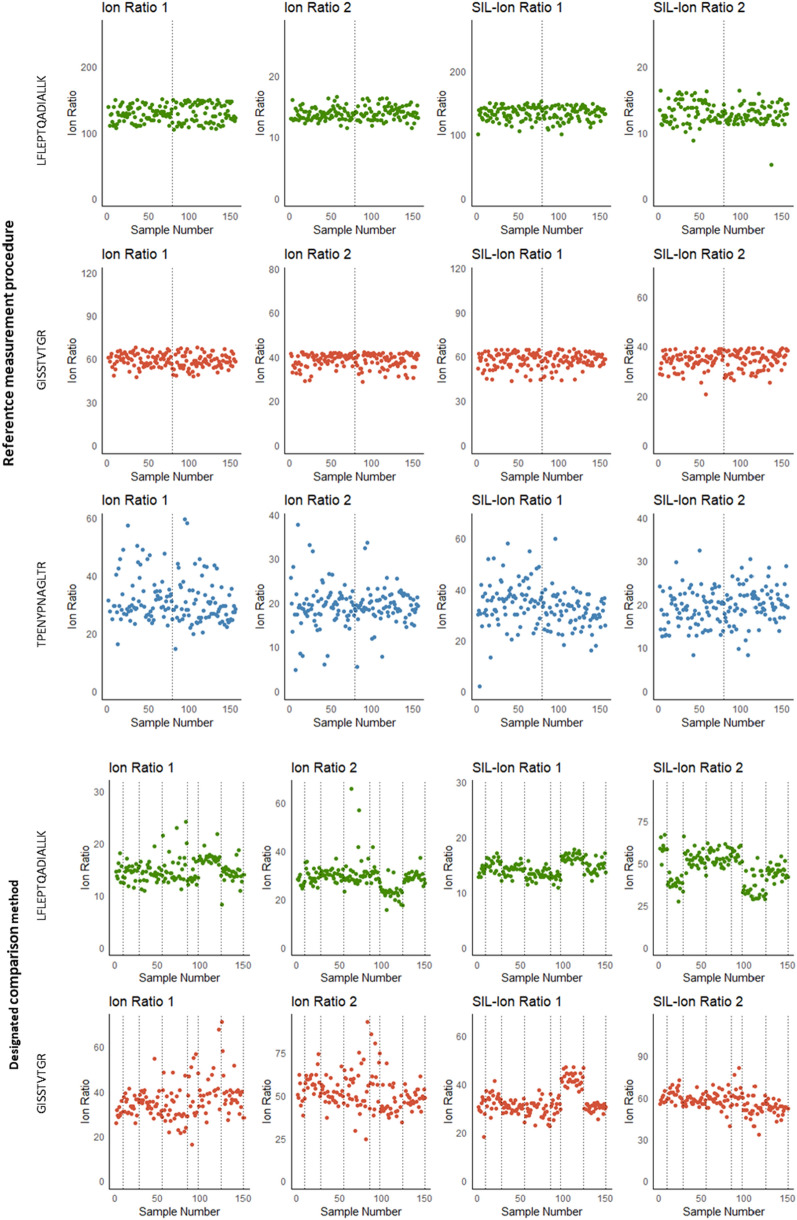
Ion ratio monitoring of the results obtained with the RMP (top panel) and the DCM (lower panel). Ion ratios were defined as the ratio between the quantifying transition and each of the qualifying transitions (Table 2). Dotted lines indicate different measurement batches, which may have occurred at different instruments (n = 4) for the DCM, resulting in inter-batch variability. However, within a batch, consistent ion ratios are observed

To further assess quality of the individual sample results, ion ratios between quantifying and qualifying transitions, internal standard areas and interpeptide agreements were monitored for both the RMP as well as the DCM. Ion ratios are affected by MS instrument settings, and the DCM was performed on four different LC–MS systems. Therefore, summary statistics of the ion ratios were calculated for each measurement batch individually. Overall, the ion ratios are consistent between endogenous peptides and internal standard peptides and CVs range between 7.3% and 12.8% for peptide LFLEPTQADIALLK of the RMP and 5.3% and 26% for the DCM (Table 5), indicating consistent measurements throughout each batch. The ion ratios of the individual samples are plotted in Fig. 2; no individual outliers were observed, suggesting that results are not confounded by interferences.

**Table 5:** Summary statistics of ion ratio monitoring

Reference Measurement Procedure													
		GISSTTVTGR Qualifier 2	GISSTTVTGR Qualifier 1	GISSTTVTGR-SIL Qualifier 2	GISSTTVTGR-SIL Qualifier 1	LFLEPTQADIALLK Qualifier 1	LFLEPTQADIALLK Qualifier 2	LFLEPTQADIALLK-SIL Qualifier 1	LFLEPTQADIALLK-SIL Qualifier 2	TPENYPNAGLTR Qualifier 1	TPENYPNAGLTR Qualifier 2	TPENYPNAGLTR-SIL Qualifier 1	TPENYPNAGLTR-SIL Qualifier 2
Batch 1	Average ion ratio	39	59	34	57	128	14	133	13	34	19	35	19
	*CV (%)*	*8.0*	*7.7*	*10.4*	*9.8*	*9.0*	*7.3*	*8.1*	*12.8*	*38.1*	*27.2*	*25.3*	*22.4*
Batch 2	Average ion ratio	38	59	34	57	129	14	133	13	32	19	33	19
	*CV (%)*	*8.5*	*8.3*	*10.8*	*9.3*	*10.5*	*7.8*	*8.3*	*12.6*	*34.0*	*24.4*	*24.7*	*21.9*

Designated Comparison Method									
		GISSTVTGR Qualifier 1	GISSTVTGR Qualifier 2	GISSTTVTGR-SIL Qualifier 1	GISSTTVTGR-SIL Qualifier 2	LFLEPQADIALLK Qualifier 1	LFLEPTQADIALLK Qualifier 2	LFLEPTQADIALLK-SIL Qualifier 1	LFLEPTQADIALLK-SIL Qualifier 2
Batch 1	Average ion ratio	31	50	30	59	15	27	14	59
	*CV (%)*	*9.2*	*14.2*	*16.7*	*5.1*	*11.8*	*6.9*	*5.3*	*8.7*
Batch 2	Average ion ratio	33	56	33	61	15	29	15	45
	*CV (%)*	*11.9*	*13.3*	*13.3*	*7.9*	*10.6*	*10.2*	*8.7*	*24.6*
Batch 3	Average ion ratio	33	53	31	59	15	30	14	50
	*CV (%)*	*17.8*	*18.5*	*12.0*	*8.9*	*26.0*	*18.6*	*8.5*	*16.6*
Batch 4	Average ion ratio	34	54	31	60	15	30	14	51
	*CV (%)*	*21.2*	*19.9*	*11.7*	*9.7*	*25.1*	*18.0*	*9.0*	*15.9*
Batch 5	Average ion ratio	35	53	33	58	15	29	14	48
	*CV (%)*	*22.1*	*20.7*	*17.6*	*11.7*	*22.6*	*20.9*	*10.8*	*21.1*
Batch 6	Average ion ratio	36	52	33	57	15	29	14	47
	*CV (%)*	*22.8*	*19.6*	*16.8*	*11.8*	*21.7*	*19.3*	*10.4*	*19.8*

**Fig. 3:**
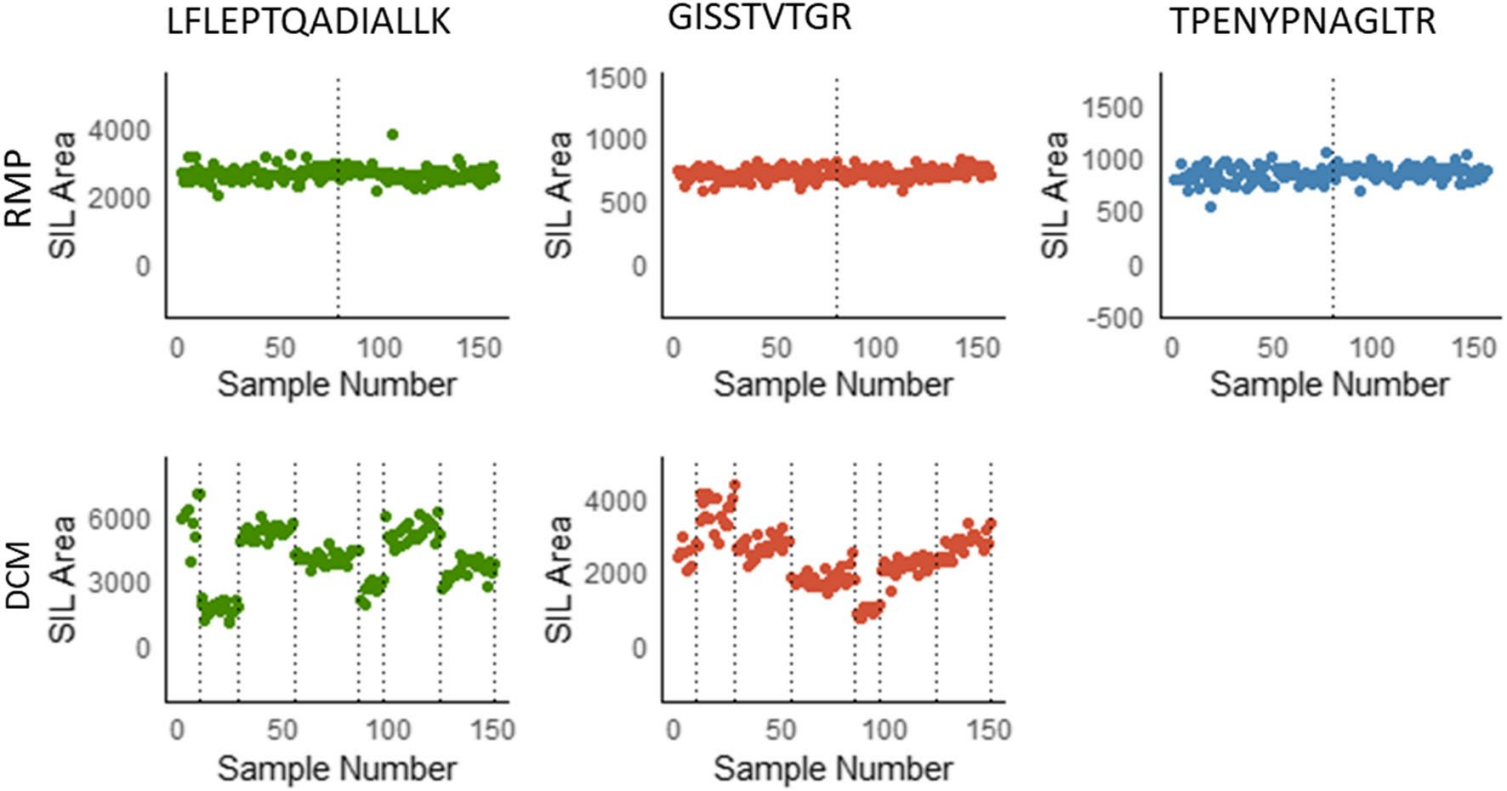
Internal standard area monitoring of the results obtained with the RMP (top panel) and DCM (lower panel). Areas from each of the measurements is plotted, and the various different batches are indicated by vertical dotted lines

A third measure of measurement quality is the stability of the internal standard. Summary statistics (mean and CV) of the signal intensities of the internal standard peptides can be found in Table 6 and Fig. 3. In Fig. 3, the observed differences in batches can be attributed to the use of multiple mass spectrometers, specifically the 6495A and 6495C triple quadrupole instruments. However, for the RMP, the variation was low, with CVs ranging between 6.9% and 10.3%. Similar CVs were observed for the DCM.

**Table 6:** Summary statistics of internal standard area monitoring

Batch	Peptide	Average signal intensity (counts)	CV (%)	n
Reference Measurement Procedure				
Batch 1	GISSTTVTGR	697	7.1	75
	LFLEPTQADIALLK	2659	8.4	75
	TPENYPNAGLTR	825	10.3	75
Batch 2	GISSTTVTGR	717	6.5	78
	LFLEPTQADIALLK	2641	8.7	78
	TPENYPNAGLTR	864	7.3	78
Designated Comparison Method				
Batch 1	GISSTTVTGR	2502	11.4	9
	LFLEPTQADIALLK	5878	16.5	9
Batch 2	GISSTTVTGR	3579	12.9	19
	LFLEPTQADIALLK	1715	19.3	19
Batch 3	GISSTTVTGR	2687	9.1	27
	LFLEPTQADIALLK	5291	5.6	27
Batch 4	GISSTTVTGR	1815	12.0	31
	LFLEPTQADIALLK	4070	6.7	31
Batch 5	GISSTTVTGR	2002	11.0	12
	LFLEPTQADIALLK	4476	14.8	12
Batch 6	GISSTTVTGR	2172	9.4	27
	LFLEPTQADIALLK	5205	8.8	27
Batch 7	GISSTTVTGR	2744	10.2	28
	LFLEPTQADIALLK	3519	12.2	28

**Fig. 4:**
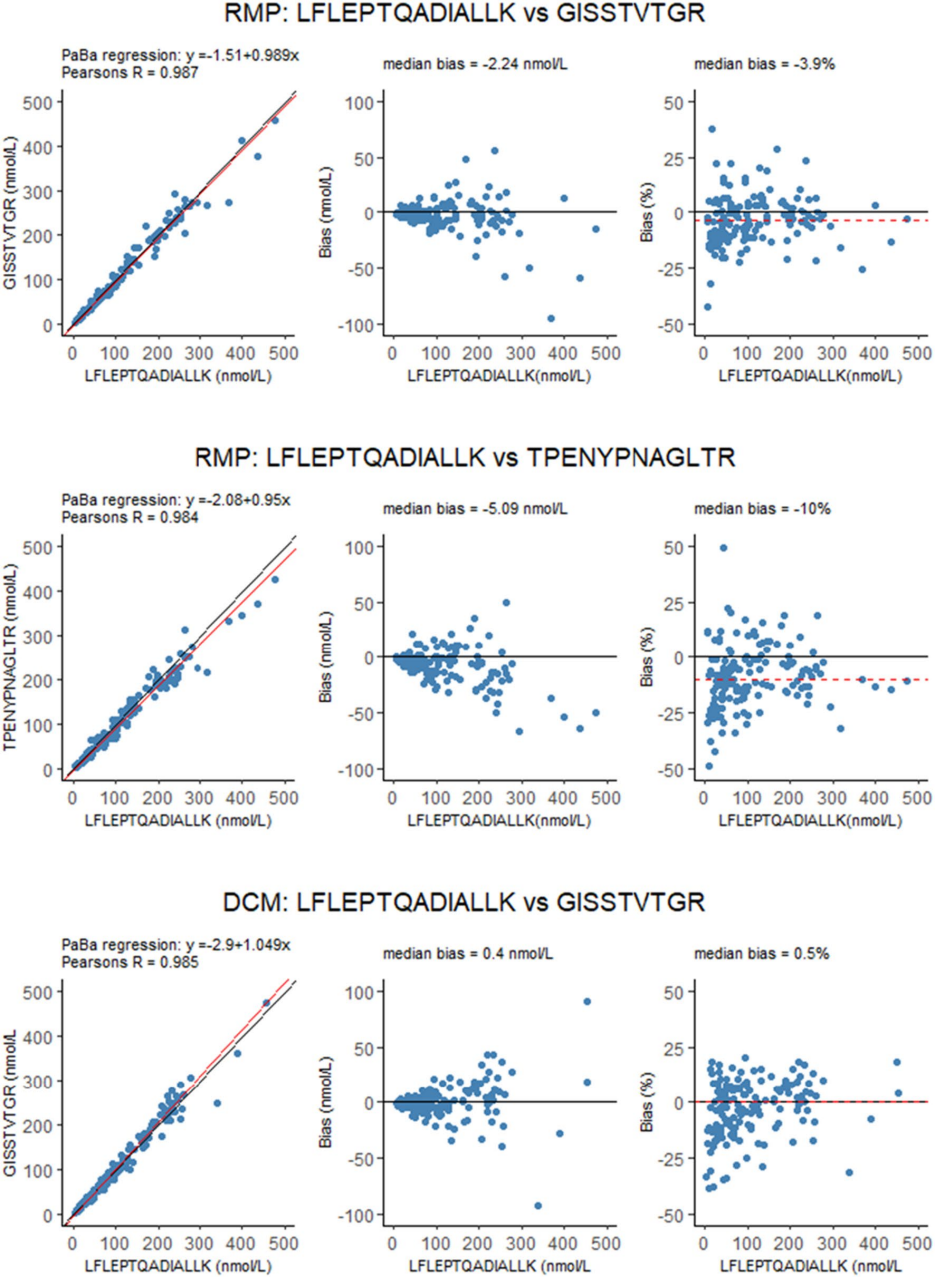
Interpeptide comparisons for both the RMP and the DCM. Passing-Bablok regression is indicated in the regression plot (red line), while the black line indicates the line of identity. Red dotted line in the bias plots indicates median (percent) bias

A final tool to assess data validity is interpeptide comparison (Fig. 4). As each of the peptides monitored should represent the same protein, results of the peptides should provide equivalent results, within analytical variation. Comparisons were made for both the RMP and the DCM and Passing-Bablok regression analysis revealed slopes of 0.989 and 0.950 between peptide LFLEPTQADIALLK and peptides GISSTVTGR and TPENYPNAGLTR, respectively for the RMP and 1.04 between peptide LFLEPTQADIALLK and GISSTVTGR for the DCM (Table 7). Correlation coefficients were also as expected, 0.987 and 0.984 for the RMP and 0.985 for the DCM. Overall, assessment of these quality measures indicated that the performance of the DCM generated valid and robust metadata, comparable to those of the RMP.

**Table 7:** Results of interpeptide comparison by Passing-Bablok non-parametric regression analysis

Test	X Peptide	Y Peptide	Intercept	Slope	Median bias (nmol/L)	Median bias (%)	Pearson's R	Mean X ± SD (nmol/L)	Median X (min – max) (nmol/L)	Mean Y ± SD (nmol/L)	Median Y (min – max) (nmol/L)	N
RMP	LFLEPTQADIALLK	GISSTVTGR	− 1.5 (− 2.7 to − 0.8)	0.989 (0.970 – 1.011)	-2.2	-3.9	0.987	112.2 ± 93.6	85 (4.4 – 475)	109.5 ± 90.9	78 (3.1 – 459	151
RMP	LFLEPTQADIALLK	TPENYPNAGLTR	− 2.1 (− 4.1 to − 1.1)	0.950 (0.916 – 0.986)	-5.1	-10	0.984	112.2 ± 93.6	85 (4.4—475)	104.3 ± 86.8	73 (4.3 – 425)	151
DCM	LFLEPTQADIALLK	GISSTVTGR	− 2.9 (− 4.2 to − 1.2)	1.049 (1.016 – 1.075)	0.4	0.5	0.985	109 + 89.4	80 (3.8 – 455)	109.9 ± 94.1	78 (2.7 – 544)	150

### Method comparison

**Fig. 5:**
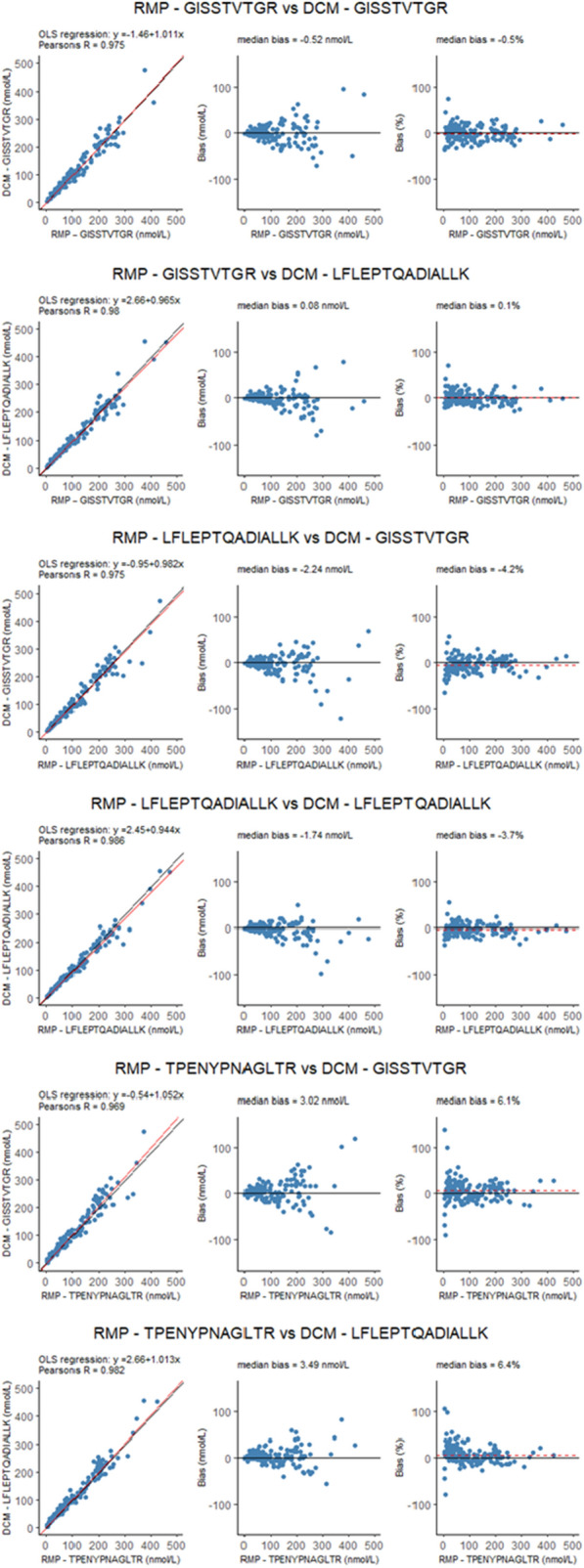
Method comparisons between results obtained with the Reference Measurement Procedure (RMP) and the Designated Comparison Method (DCM). Each of the RMP peptides is compared to each of the DCM peptides. Linear regression is used, as the X-axis represents results from the RMP, and is indicated in the regression plots (red line), while the black lines indicate the line of identity. Red dotted line in the bias plots indicated median (percent) bias

A direct method comparison was performed between the RMP and the DCM. The LoQ of the RMP is 3.8 nmol/L and results below this threshold were removed from the analysis per peptide. Ordinary least squares linear regression was employed for the method comparison, as the RMP, which is the anchor point, was included on the x-axis. Results of the method comparisons for each of the peptides are summarized in Table 8 and shown in Fig. 5. Median slope for the comparisons was 0.997, with slopes ranging between 0.944 and 1.052. Negligible and non-significant intercepts were observed. Biases ranged between -2.2 nmol/L and 3.5 nmol/L, with a median bias of 0.2 nmol/L (0.2%). Correlation coefficients are all ≥ 0.975 except for the comparison between RMP peptide TPENYPNAGLTR and DCM peptide GISSTVTGR (r = 0.969). However, peptide TPENYPNAGLTR is included as qualifying peptide, only to confirm quantitative results. The observed variation between the RMP and the DCM are within expected analytical variation, therefore the performance of the RMP and the DCM is equivalent.

**Table 8:** Results of method comparison

RMP Peptide	DCM Peptide	Intercept	Slope	Median bias (nmol/L)	Median bias (%)	Pearson's R	Mean X ± SD (nmol/L)	Median X (min – max) (nmol/L)	Mean Y ± SD (nmol/L)	Median Y (min – max) (nmol/L)	n
GISSTVTGR	GISSTVTGR	− 1.5 (− 7.6 – 4.6)	1.011 (0.937 – 1.082)	− 0.5	− 0.5	0.975	110.2 ± 90.7	80 (4.3 – 459)	109.9 ± 94.1	78 (2.7 – 544)	150
GISSTVTGR	LFLEPTQADIALLK	2.7 (− 1.6 – 6.6)	0.965 (0.910 – 1.026)	0.1	0.1	0.98	110.2 ± 90.7	80 (4.3 – 459)	109 ± 89.4	80 (3.8 – 455)	150
LFLEPTQADIALLK	GISSTVTGR	− 0.9 (− 6.2 – 5.7)	0.982 (0.899 – 1.052)	− 2.2	− 4.2	0.975	112.2 ± 93.6	85 (4.4 – 475)	109.2 ± 94.2	76 (1.9 – 544)	151
LFLEPTQADIALLK	LFLEPTQADIALLK	2.4 (− 0.4 – 5.9)	0.944 (0.899 – 0.982)	− 1.7	− 3.7	0.986	112.2 ± 93.6	85 (4.4 – 475)	108.3 ± 89.5	80 (3.5 – 455)	151
TPENYPNAGLTR	GISSTVTGR	− 0.5 (− 6.7 – 6.0)	1.052 (0.963 – 1.141)	3	6.1	0.969	103.6 ± 86.9	72 (4.3 – 425)	108.5 ± 94.3	76 (1.0 – 544)	152
TPENYPNAGLTR	LFLEPTQADIALLK	2.7 (-1.3 – 7.0)	1.013 (0.951 – 1.067)	3.5	6.4	0.982	103.6 ± 86.9	72 (4.3 – 425)	107.6 ± 89.6	78 (1.9 – 455)	152

## Discussion

With the inclusion of Lp(a) as a relevant genetic risk factor in clinical guidelines on cardiovascular risk management, and Lp(a) lowering therapies in phase III clinical studies, accurate measurement of Lp(a) in medical laboratories is more urgent than ever. As Lp(a) and its characteristic apolipoprotein apo(a) are highly heterogeneous, measurements of apo(a) in molar units are recommended. To ensure an unequivocally defined measurand, a new RMS based on SI-traceable primary calibrators and an LC–MS based RMP is in preparation by the IFCC WG APO-MS [8, 25]. However, until the full RMS including the calibration is in place, a method is needed to pave the way for the transition from mass units to molar units with IVD manufacturers.

To this end, we developed a semi-automated MS-based DCM for apo(a), with equivalent performance compared to the more laborious RMP. The DCM is based on the quantitative bottom-up proteomics strategy in which peptides are quantified as surrogates of proteins. Therefore, stringent peptide selection is imperative [17], as has been described for the RMP [10]. In the RMP three proteotypic peptides are quantified: GISSTVTGR, LFLEPTQADIALLK and TPENYPNAGLTR. Peptide TPENYPNAGLTR may be prone to a genetic variant, which occurs in ~ 6% of individuals of African descent and may only serve as qualifying peptide to confirm obtained quantitative results [10]. For the DCM, monitoring of two peptides suffices and the best performing peptides in terms of precision (GISSTVTGR and LFLEPTQADIALLK) have been included in the DCM.

Analytical specificity of the method is (among others) achieved through the selection of specific transitions. For the RMP, common transitions were selected that could be monitored in all three calibration laboratories of the Lp(a) calibration network, using different LC/MS-instruments. However, the DCM was developed in a single calibration laboratory. Therefore, for the RMP the analytically less specific b2 fragments were included as qualifying fragments, whereas the b3 fragments could be selected for the DCM (Table 2).

The native human serum-based calibration used in the RMP has a temporary character, as an SI-traceable calibration is envisioned. Therefore, the RMP makes use of only a single calibrator for apo(a). Contrarily, the DCM employs five native human serum calibrators. The calibrators of both the RMP and the DCM were indirectly value assigned for their apo(a) concentration using an immunoassay traceable to WHO-IFCC reference material SRM2B.

A third major difference between the RMP and the DCM is the digestion procedure. For the RMP, peptide-based calibration is envisioned to achieve traceability to the International System of measures [8], which requires complete, equimolar digestion. Moreover, the RMP is a multiplexed procedure, not only intended for quantitation of apo(a), but also other apolipoproteins. To enhance the digestion efficiency and reach stable digestion plateaus for all the peptides monitored in the RMP, a pre-digestion using Lys-C was introduced [10]. This step is, however, costly, both timewise and monetarily. As the DCM will not use primary reference materials for calibration, but native human serum calibrators (secondary reference materials), only stable digestion is required, as long as the calibrators behave the same compared to the human serum samples. Intermediate imprecision of the RMP and the DCM are equivalent and fulfill the predefined analytical performance specifications of 50% of the TEa (ranging between 21.3% and 7.8% at Lp(a) concentrations of 5.0 nmol/L and 344 nmol/L, respectively). Results of the method comparison also indicate equivalence of results. Therefore, it may be concluded that the method may be used as a higher order DCM for the apo(a) RMP.

An essential step for the increased throughput in the DCM is the application of a liquid handling robot for semi-automation of the sample preparation method. As we transition from the old RMS to the new RMS, the IVD-industry will be directed through the utilization of the DCM for apo(a). LC–MS technology is, currently, not yet available on consolidated and robust clinical chemistry analyzers. Therefore, sample preparation must be performed in ‘batch-mode’. Batches of 96 are ideally suited for semi-automation using liquid handlers, providing more consistent test results combined with a more facile and less error-prone procedure. While the RMP was not yet implemented on a liquid handling platform, the semi-automated DCM is, which enables higher sample throughput.

Both the RMP and the DCM are LC–MS based procedures. LC–MS provides significant advantages of traditional immunoassays for the quantitation of heterogeneous proteins [26]. Specifically, the detection method allows quantitation of molecularly defined measurands [17], is antibody independent, and may generate intrinsic metadata to determine validity of individual results [27]. The measurands as defined in the DCM are molar concentrations of peptides GISSTVTGR and LFLEPTQADIALLK, reflecting apo(a) in human serum, and are by definition independent of apo(a) KIV-2 repeats. An important disadvantage of immunoassays is their vulnerability for interferences generated by non-specific binding or masking of the target protein. In the RMP and DCM LC–MS procedures both IQC and intrinsic metadata were assessed to ensure individual results generated are correct. In particular, ion ratios between quantifying and qualifying transitions, stability of the internal standard signal and interpeptide agreements may reveal variation, and monitoring of these parameters is therefore recommended (CLSI C64)[28], and was performed.

In the direct method comparison performed between the RMP and the DCM equivalent results were obtained. Therefore, the DCM may be used as a more facile method compared to the RMP in the establishment of standardization of Lp(a) tests.

**Fig. 6:**
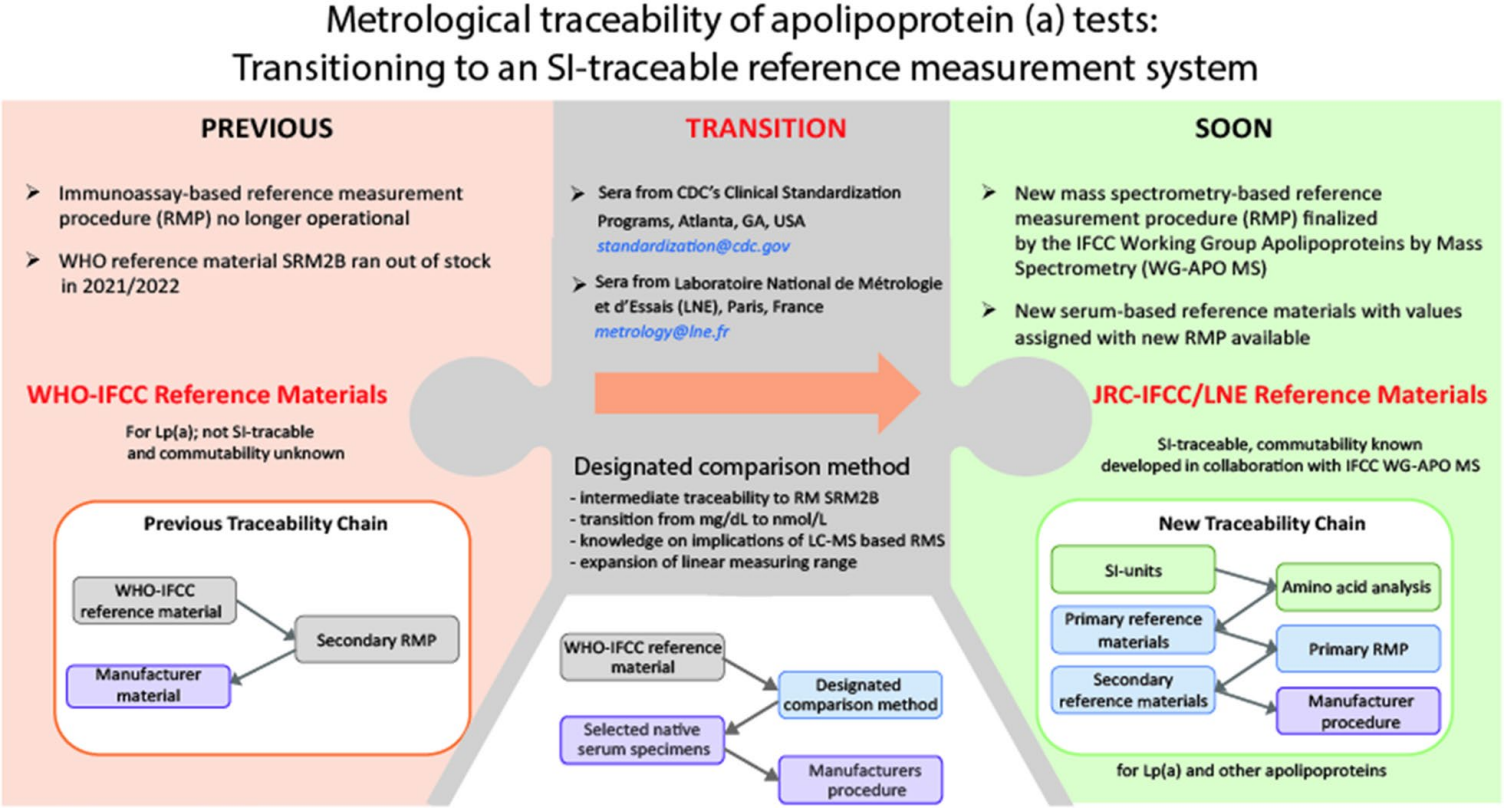
Schematic representation of the transition from the former ELISA-based Reference Measurement System (NWLRC, Washington, USA) to an MS-based Reference Measurement System [8] that is in development. The here presented Designated Comparison Method (DCM) will have a key role in the transition phase, in which manufacturers can maintain SRM2B traceability, while abolishing inaccuracy due to multiple misinterpretations around the Lp(a) metrics

Current Lp(a) tests may or may not be traceable to the previous RMS, that consisted of a reference material that is not SI-traceable, but value assigned in nmol/L (SRM2B), and a KIV-2 independent ELISA-based RMP (Fig. 6) [29, 30]. However, this RMS is no longer available, as the material has run out of stock and the RMP is no longer operational. The new LC–MS based RMS is still in development, and therefore a 2-phased process for Lp(a) re-standardization is advocated by the IFCC WG APO-MS: first, the transition from mass to molar units should be accomplished, to overcome the unavailability of an operational RMS for apo(a). Secondly, the final step for Lp(a) re-standardization should be realized when the entire MS-based RMS is in place, including the peptide-based calibration. As the results of the RMP and the DCM are equivalent, the DCM can be used as a provisional anchor to guide IVD-manufacturers with Lp(a) re-standardization to molar units. Specifically, a method comparison program is being built in which well-characterized samples are quantified using both the DCM and the manufacturer’s method. The samples will cover a wide Lp(a) concentration range, as well as a variety of known apo(a) KIV-2 repeats. Through evaluation of the obtained data, manufacturers of Lp(a) immunoassays will be able to obtain data on three important aspects (Fig. 6): 1. Recommendations on their current level of traceability to previous reference material SRM2B in nmol/L, including the level of KIV-2 dependency of their test. 2. Recommendations on the effects of sample dilution in samples containing high apo(a) concentrations that are above the linear measuring range. And 3. Information on the expected implications of the transition from the previous ELISA-based RMS to the new LC–MS based RMS.

## Conclusion

We here present a semi-automated LC–MS based DCM for apo(a)/Lp(a) that is apo(a) KIV-2 independent, fulfills predefined analytical performance specifications and provides equivalent results compared to the IFCC-endorsed apo(a) RMP [10]. The Lp(a) DCM will be utilized to guide IVD-manufacturers to make the necessary transition from flawed mass units to molar units. While the transition to well defined apo(a) measurands and their expression in molar units is a long and challenging road, the apo(a) transition from mass to molar is an essential first step to document the relation between former and new RMP, to solve the metrological misunderstandings around Lp(a) metrics and to enable more refined cardiovascular patient management.

## Data Availability

The datasets generated and analyzed during the current study are available from the corresponding author on reasonable request.

## Abbreviations

ABC: Ammonium bicarbonate
Apo: Apolipoprotein
APS: Analytical Performance Specifications
BD: Becton Dickinson
CLSI: Clinical and Laboratory Standards Institute
CV: Coefficient of Variation
CVi: Intra-individual Coefficient of Variation
CVg: Inter-individual Coefficient of Variation
DCM: Designated Comparison Method
DOC: Sodium Deoxycholate
ELISA: Enzyme-Linked Immunosorbent Assay
FA: Formic Acid
HLB: Hydrophilic-Lipophilic Balance
IAM: Iodoacetamide
IQC: Internal Quality Control
IFCC: International Federation of Clinical Chemistry and Laboratory Medicine
IFCC WG APO-MS: International Federation of Clinical Chemistry and Laboratory Medicine Working Group for Standardization of Apolipoproteins by Mass Spectrometry
ISO: International Organization for Standardization
IVD: In vitro diagnostics
KIV-2: Kringle IV type 2
LC-MS: Liquid Chromatography—Mass Spectrometry
Lp(a): Lipoprotein(a)
MeOH: Methanol
RMP: Reference Measurement Procedure
RMS: Reference Measurement System
SI: International System of Units
SIL: Stable Isotope Labeling
SRM2B: Standard Reference Material 2B
TCEP: Tris(2-carboxyethyl)phosphine
TEa: Total Allowable Error
TRIS: Tris(hydroxymethyl)aminomethane
